# The Association between Meteorological Parameters and Aneurysmal Subarachnoid Hemorrhage: A Nationwide Analysis

**DOI:** 10.1371/journal.pone.0112961

**Published:** 2014-11-13

**Authors:** Pui Man Rosalind Lai, Hormuzdiyar Dasenbrock, Rose Du

**Affiliations:** 1 Department of Neurosurgery, Brigham and Women's Hospital, Boston, Massachusetts, United States of America; 2 Harvard Medical School, Boston, Massachusetts, United States of America; St Michael's Hospital, University of Toronto, Canada

## Abstract

Prior research has suggested that regional weather patterns impact the risk of rupture of cerebral aneurysms, but the findings in the literature have been inconsistent. Furthermore, no nationwide analysis to date has examined the association between meteorological factors and the post-procedural outcomes of patients after the treatment for ruptured cerebral aneurysms. The purpose of this study was to use a nationwide sample to analyze the association between specific meteorological parameters—temperature, precipitation, sunlight, and humidity—and hospital admission rate for and outcome after aneurysmal subarachnoid hemorrhage. Patients were identified using the Nationwide Inpatient Sample (2001–2010): Those with an ICD-9 diagnosis code for subarachnoid hemorrhage and a procedural code for aneurysm repair were included. Climate data were obtained from the State of the Climate Report 2010 released by the National Climatic Data Center. Multivariate regression models were constructed to analyze the association between average state monthly temperature, precipitation, and percent possible sunlight, as well as relative morning humidity and both monthly hospital admission rate, adjusted for annual state population in millions, and in-hospital mortality. 16,970 admissions were included from 723 hospitals across 41 states. Decreased daily sunlight and lower relative humidity were associated with an increased rate of admission for ruptured cerebral aneurysms (*p<0.001*), but had no association with differential inpatient mortality. No significant changes in these observed associations were seen when multivariate analyses were constructed. This is the first nationwide study to suggest that decreased sunlight and lower relative humidity are associated with admission for ruptured cerebral aneurysms. While it has been postulated that external atmospheric factors may cause hormonal and homeostatic changes that impact the risk of rupture of cerebral aneurysms, additional research is needed to confirm and further understand these relationships.

## Introduction

Aneurysmal subarachnoid hemorrhage (SAH) is believed to be a largely unpredictable, spontaneous event. However, the formation and rupture of cerebral aneurysms has been shown to be associated with multiple factors, including the characteristics of the aneurysm, the age of the patient, blood pressure, and changes in body temperature [1], [2]. Regional weather patterns and seasonal changes have also been hypothesized to impact the risk of rupture of cerebral aneurysms. Several studies have suggested that hospital admission for aneurysmal subarachnoid hemorrhage is associated with variations in temperature, atmospheric pressure, humidity, and the lunar cycle [3]–[5]. Moreover, associations between meteorological parameters and cerebral aneurysm rupture have also been observed to vary by sex [6], [7]. However, the findings in the literature have been inconsistent: other studies have suggested that subarachnoid hemorrhage does not vary with season or temperature [8], [9].

Although prior retrospective studies have analyzed the association between weather patterns and the risk of cerebral aneurysm rupture in patients treated at a single hospital or a limited geographic area, few large-population or nationwide studies have found an association between meteorological factors and aneurysmal subarachnoid hemorrhage. Furthermore, no study to date has analyzed the association between weather patterns and the post-procedural outcomes of patients treated for ruptured cerebral aneurysms. This is the first nationwide analysis to investigate the association between meteorological parameters—average daily temperature, precipitation, sunlight, and morning humidity—and hospital admission for, as well as outcomes (in-hospital mortality) after aneurysmal subarachnoid hemorrhage.

## Methods

### Database

Data were extracted from the Nationwide Inpatient Sample (NIS, Healthcare Cost and Utilization Project, Agency Healthcare Research and Quality) for the years 2001 to 2010. The NIS is the largest all-payer longitudinal inpatient care database in the United States, consisting of approximately 8 million annual hospitalizations. All discharges from sampled hospitals (from across 41 states) are included in the NIS, which is an approximately 20% stratified sample of American non-federal hospitals. The NIS contains data about diagnoses, procedures, and hospital characteristics to allow for analysis of national trends in health care outcomes.

### Inclusion Criteria and Outcome Measures

Diagnostic codes from the International Classification of Disease, 9^th^ Revision, Clinical Modification (ICD-9-CM) were used to identify patients with ruptured cerebral aneurysms. Patients were included if they had a diagnosis code for subarachnoid hemorrhage (ICD-9-CM 430) or intracerebral hemorrhage (ICD-9-CM 431) and at least one procedural code for aneurysm repair, by “clipping of aneurysm” (ICD-9-CM 39.51), “endovascular repair or occlusion” (ICD-9-CM 39.72), or “other repair of aneurysm” (ICD-9-CM 39.79).

The endpoints evaluated were state-adjusted hospital admission rate and in-hospital mortality. State-adjusted hospital admission rate was normalized by calculating the total number of patients identified by hospital state and admission month, divided by the state population in millions. In-hospital mortality was normalized by dividing the number of in-hospital deaths per state by the number of admissions per state. Hospital state, admission month and in-hospital mortality are directly encoded in the NIS database. Annual state population was obtained from the 2000–2010 State Characteristics Intercensal Population Estimates File, US Census Bureau, Population Division (http://www.census.gov/popest/data/intercensal/index.html) and was used to adjust for state hospital admission.

Climate data was obtained from the State of the Climate Report 2010 released by the National Climatic Data Center (http://www.ncdc.noaa.gov/). The report outlines the climatic conditions at major weather observing stations in all 50 states, which includes data for monthly temperature (degree Fahrenheit), monthly precipitation (inches), percent possible sunlight, and relative morning humidity. Percent possible sunlight is defined as the total time for sunshine to reach the surface of the earth, expressed as a percentage of the maximum sunlight possible from sunrise to sunset with clear sky conditions. Average relative morning humidity is a percentage of the amount of moisture in the air compared to the maximum potential moisture the air can hold at the same temperature and pressure. The monthly values of all observing stations in each state were averaged across all years to generate averaged, independent monthly data across each state for all climate variables.

### Statistical Analysis

Statistical analyses were performed using STATA 12.0 (StataCorp LP, College Station, Texas), and probability values were considered statistically significant if *p*<0.05. Multivariable linear regression analysis was performed to evaluate the association between annual average monthly state temperature, precipitation, percent possible sunlight, and relative morning humidity on annual state-adjusted hospital monthly admission rate and in-hospital mortality rate. Subsequent multivariate regression models were constructed after patients were also stratified by sex, to evaluate if sex modifies any potential associations between hospital admission or outcomes and weather.

## Results

### Study Population

16,970 patients with SAH who underwent surgical clipping or endovascular coiling to repair a cerebral aneurysm were identified from 723 hospitals across 41 states (Table 1). 67.8% (*n* = 11,484) of patients included were females. The median age for all patients [interquartile range (IQR)] was 53 (34–72), and for females and males were 54 (35–73) and 51 (33–69) respectively. 6933 (40.9%) and 7325 (43.2%) patients underwent endovascular coiling only and surgical clipping only respectively. The in-hospital mortality for the patient population was 2309 (13.6%). The mortality was 1618 (14.1%) in females and 691 (12.7%) in males. The median [IQR] hospital length of stay was 17 (3–31) days.

**Table 1:** Demographics of 16, 970 patients with SAH who underwent surgical clipping and/or endovascular coiling.

Age (median years, interquartile range)	53 (34–72)
Females	11,484 (67.8%)
Treatment: Clipping only	6933 (40.9%)
Treatment: Coiling only	7325 (43.2%)
Mortality	2309 (13.6%)
Hospital length-of-stay (median days, interquartile range)	17 (3–31)

### Multivariate Analyses

Multivariate regression was utilized to analyze the association between average monthly state temperature, precipitation, daily percent possible sunlight, and relative morning humidity on state-adjusted hospital admissions for and mortality during hospitalization for aneurysmal SAH, adjusted by sex (Tables 2 and 3). Higher daily percent sunlight and greater average morning humidity were associated with a decreased rate of state population adjusted hospital admission (*p*<0.001); however, no significant differences in mortality were seen. Greater precipitation was associated with reduced in-hospital mortality (*p* = 0.001). Temperature was not found to be associated with significantly different rates of admission or in-hospital mortality. No changes in the statistical significance of parameters were found when multivariate analyses were constructed without sex as a covariate (data not shown).

**Table 2:** Multivariate analysis evaluating the association between meteorological parameters by state and annual state population-adjusted hospital admission rate for aneurysmal subarachnoid hemorrhage, adjusted for sex.

State-Adjusted Hospital Admission	Coefficient	95% Confidence Interval	p
Average Daily Temperature	0.012	(0.002, 0.02)	0.021
Average Daily Precipitation	−0.044	(−0.2, 0.07)	0.426
Average Percent Daily Sunlight	**−0.061**	**(−0.08, −0.04)**	**<0.001**
Average Morning Humidity	**−0.041**	**(−0.05, −0.03)**	**<0.001**

**Table 3:** Multivariate analysis evaluating the association between meteorological parameters by state and in-hospital mortality after aneurysmal subarachnoid hemorrhage, adjusted for sex.

In-hospital Mortality	Coefficient	95% Confidence Interval	p
Average Daily Temperature	0.0007	(−0.003, 0.002)	0.158
Average Daily Precipitation	**−0.017**	**(−0.03, −0.007)**	**0.001**
Average Percent Daily Sunlight	−0.001	(−0.003, 0.0003)	0.119
Average Morning Humidity	−0.004	(−0.002, −0.0008)	0.512

## Discussion

The degree to which the rupture of cerebral aneurysms is impacted by variations in season or weather has been widely debated. Many institutional or regional retrospective studies have examined the relationship between climate and aneurysmal subarachnoid hemorrhage, but the findings in the literature have been inconsistent. Prior research has suggested that ambient temperature, precipitation, sunlight, average humidity, and the lunar cycle may all be associated with an increased risk of rupture of cerebral aneurysms [4], [5], [10]–[13]. On the other hand, others have found no associations between weather patterns and subarachnoid hemorrhage [3], [8], [9]. One recent large meta-analysis review of the literature found SAH to be associated with the winter and January [14]. In the analysis, multiple studies reported associations with temperature and humidity, although the directions of effects were conflicting due to the heterogeneity of the population pool. To date, the climatic effects on spontaneous SAH continue to be unclear and conflicting.

In this study, 16,970 patients from 723 hospitals across 41 US states over a 10-year period who presented with aneurysmal subarachnoid hemorrhage were analyzed. Four specific meteorological parameters were examined—temperature, precipitation, sunlight, and humidity—to evaluate how these factors are associated with admission rates for and outcomes after aneurysmal subarachnoid hemorrhage. After adjusting for state population, greater sunlight and higher average morning humidity were found to be associated with decreased rate of hospital admission for ruptured cerebral aneurysms. The increased admission with decreased sunlight is consistent with prior studies demonstrating increased admission with winter and January.

There are few reports studying the correlation of aneurysmal SAH and sunlight exposure. Neidert *et al.* recently reported no association between hourly sunlight and incidence of aneurysmal subarachnoid hemorrhage [15]. However, there have been prior studies suggesting a distinct latitudinal pattern for subarachnoid hemorrhage occurrence with progressively decreasing rates from north to south [16]–[18]. Furthermore, multiple studies have associated summer months with lower rates of subarachnoid hemorrhage [14], [19]. These phenomena have previously been postulated to be associated with hours of sunshine [20]. Sunlight exposure have also been correlated previously with cerebral infarction [21] as well as with the rupture of abdominal aortic aneurysms [22].

It has been postulated that sunlight may impact blood pressure, which ultimately affects the risk of aneurysm rupture. There are new evidences suggesting seasonal blood pressure to be associated with daylight hours [23]. Sunlight exposure has been shown to alter blood pressure through the effects of UV light on vitamin D and parathyroid hormone status, stimulating changes in vascular smooth muscle and intracellular calcium, adrenergic responsiveness, and endothelial function [24], [25]. Vitamin D deficiency is associated with high blood pressure and the prevalence of the deficiency has seasonal as well as geographic variation based on sunlight exposure [26]. Thus, it is biologically plausible that the association between decreased sunlight hours and greater hospital admissions of cerebral aneurysms may be at least partially attributable to the effects of Vitamin D on blood pressure. Moreover, blood pressure is a known independent risk factor associated with both formation and rupture of cerebral aneurysms [27], [28].

Variations in blood pressure have been proposed in the literature to explain the bimodal circadian distribution of SAH, with initial peak during morning hours and a second peak in the late afternoon [29]. Thus, the relationship between sunlight and aneurysm rupture may also be explained through its indirect effect on blood pressure via the circadian rhythm. Given the model of monthly admission used in this study and lack of specific blood pressure data in NIS, we were unable to account for blood pressure as a covariate. Further studies in investigating this relationship would be important to further our understanding on the association of sunlight with aneurysmal rupture.

The association of sunlight exposure and SAH has also been attributed to the regulation of serotonin by the light/dark cycle. Serotonin in CSF has been reported to fluctuate with both daylight exposure and seasonal variations, with peaks in the spring and troughs in the fall [30], [31]. It has been postulated that the changes in serotonin level affect the sensitivity of cerebral vessels [16], and subsequently, influences the rupture of cerebral aneurysms. Furthermore, sunlight exposure may also affect patient behaviors, such as tobacco smoking and exercise, which may also impact the risk of aneurysm rupture [32].

Studies have previously reported no association between average humidity and spontaneous SAH [3], [11], [15], [33]–[35]. One systematic review and meta-analysis found a total of 15 studies on the association of relative humidity and SAH incidence. Among the studies, a significant relationship between SAH and humidity was found in three studies [14], [36]. In our analysis, lower relative morning humidity was found to be associated with increased rate of hospital admission for aneurysmal subarachnoid hemorrhage. One study found that decreased relative humidity and air pressure increase insensible water loss and blood viscosity [37]. Arterial wall shear stress is directly proportional to velocity of blood flow and blood viscosity, potentially increasing the risk of aneurysm rupture. Cigarette smoking has been proposed to have a similar mechanism of increasing the risk of cerebral aneurysm rupture: increasing wall shear stress through greater blood viscosity and volume [38]. Thus, it is possible that low relative humidity may have a transient effect on blood viscosity.

While many previous studies have examined the relationship between ambient temperature and aneurysmal hemorrhage, the data has been conflicting. While some studies have reported no association between temperature and SAH [9], [15], [34]–[36], others have found a correlation of aneurysmal hemorrhage with low or extreme temperatures [2], [5], [7], [25], [33], [39]. In this study, average monthly temperature was not found to be associated with a differential rate of hospital admission for ruptured cerebral aneurysms. It has been postulated that cold temperature exposure activates the sympathetic nervous system, increasing blood pressure and resulting in increased risk of aneurysm rupture. Similar to sunlight exposure, temperature can also affect behaviors, including exercise, tobacco and alcohol consumption that may influence the risk of aneurysm rupture [40]. Although temperature is influenced by many factors, it is correlated with sunlight. It can also be postulated that the previously reported association between rupture of cerebral aneurysms and temperature may be at least partially attributable to differential sunlight exposure. In this study, sunlight and temperature were analyzed separately and only sunlight, but not atmospheric temperature, was found to be associated with hospital admission rates.

In this study, greater precipitation was found to be associated with significantly reduced in-hospital mortality after, but not with different admission rates for subarachnoid hemorrhage. This may be related to the decreased vitamin D levels that are associated with increased precipitation and associated decreased sunlight. Vitamin D has been shown to affect the immune system and to increase the production of antimicrobial peptides such as cathelicidins [41]. Lower cathelicidin levels have been shown to be correlated with increased mortality in patients with community-acquired pneumonia [42] and low vitamin D levels have been shown to be associated with increased mortality in critically ill patients [43]. In addition, increased precipitation may be associated with increased depression and seasonal affective disorders [44] that may lead to physical and emotional stress or health-related behaviors such as propensity to seek care that may affect outcome.

Prior research has suggested that the association between weather patterns and cerebral aneurysm rupture may vary by sex. Some studies have reported an association between admission for subarachnoid hemorrhage and seasonal variations in females, while others have only made this observation in males [3], [45]. To evaluate whether there is any associations between meteorological parameters and cerebral aneurysm rupture differ based on sex, separate regression analysis were constructed with and without sex as a covariate. Sex was not found to change the observed associations between sunlight or humidity and hospital admission for aneurysmal subarachnoid hemorrhage.

There are many important limitations of this study. Weather patterns were examined in this study by state, but there may be large variations in meteorological parameters within given states. Moreover, average monthly values were utilized for the individual meteorological factors, but likewise weather may change substantially within a specific month. Prior analyses have suggested that daily changes in weather may be associated with aneurysmal subarachnoid hemorrhage [33], [39]; however, daily variations could not be examined as the specific day of admission is not available in the NIS. Barometric pressure is an additional meteorological parameter that has been previously reported to be associated with the rupture of cerebral aneurysms [27], but this data was not available from the State of the Climate Report. Coding inaccuracies are a potential concern for any study based on ICM-9-CM identifiers. Furthermore, this study population was limited to those who underwent procedural treatment for cerebral aneurysm repair, but did not include patients who died before intervention.

Nonetheless, the NIS is the largest all-payer database in the United States, and few large sample studies have found a relationship between weather patterns and cerebral aneurysm rupture. Moreover, the NIS provides a very large sample of patients from a large geographic area who presented across a decade, allowing for a comprehensive analysis of the association between meteorological parameters and subarachnoid hemorrhage.

## Conclusions

This is the first nationwide retrospective study to associate sunlight exposure and humidity with the risk of cerebral aneurysm rupture. Greater precipitation was associated with reduced in-hospital mortality. The associations of external climatic factors with aneurysmal subarachnoid hemorrhage may be explained by homeostatic regulation, hormonal fluctuations and changes in human behavior, although further investigation is needed to elucidate these connections. Further research is needed to confirm these findings and further understand the pathophysiology of these relationships between climate and cerebral aneurysms.
